# A simple machine learning model for the prediction of acute kidney injury following noncardiac surgery in geriatric patients: a prospective cohort study

**DOI:** 10.1186/s12877-024-05148-1

**Published:** 2024-06-25

**Authors:** Xiran Peng, Tao Zhu, Qixu Chen, Yuewen Zhang, Ruihao Zhou, Ke Li, Xuechao Hao

**Affiliations:** 1 Department of Anesthesiology, National Clinical Research Center for Geriatrics, West China Hospital, Sichuan University, Chengdu, 610041 China; 2 Research Unit for Perioperative Stress Assessment and Clinical Decision, Chinese Academy of Medical Sciences (2018RU012), West China Hospital, Sichuan University, Chengdu, China; 3 Center of Statistical Research, School of Statistics, Southwestern University of Finance and Economics, Chengdu, China; 4 Joint Lab of Data Science and Business Intelligence, School of Statistics, Southwestern University of Finance and Economics, Chengdu, China

**Keywords:** Acute kidney injury, Noncardiac surgery, Geriatric assessment, Risk assessment, Machine learning

## Abstract

**Background:**

Surgery in geriatric patients often poses risk of major postoperative complications. Acute kidney injury (AKI) is a common complication following noncardiac surgery and is associated with increased mortality. Early identification of geriatric patients at high risk of AKI could facilitate preventive measures and improve patient prognosis. This study used machine learning methods to identify important features and predict AKI following noncardiac surgery in geriatric patients.

**Methods:**

The data for this study were obtained from a prospective cohort. Patients aged ≥ 65 years who received noncardiac surgery from June 2019 to December 2021 were enrolled. Data were split into training set (from June 2019 to March 2021) and internal validation set (from April 2021 to December 2021) by time. The least absolute shrinkage and selection operator (LASSO) regularization algorithm and the random forest recursive feature elimination algorithm (RF-RFE) were used to screen important predictors. Models were trained through extreme gradient boosting (XGBoost), random forest, and LASSO. The SHapley Additive exPlanations (SHAP) package was used to interpret the machine learning model.

**Results:**

The training set included 6753 geriatric patients. Of these, 250 (3.70%) patients developed AKI. The XGBoost model with RF-RFE selected features outperformed other models with an area under the precision-recall curve (AUPRC) of 0.505 (95% confidence interval [CI]: 0.369–0.626) and an area under the receiver operating characteristic curve (AUROC) of 0.806 (95%CI: 0.733–0.875). The model incorporated ten predictors, including operation site and hypertension. The internal validation set included 3808 geriatric patients, and 96 (2.52%) patients developed AKI. The model maintained good predictive performance with an AUPRC of 0.431 (95%CI: 0.331–0.524) and an AUROC of 0.845 (95%CI: 0.796–0.888) in the internal validation.

**Conclusions:**

This study developed a simple machine learning model and a web calculator for predicting AKI following noncardiac surgery in geriatric patients. This model may be a valuable tool for guiding preventive measures and improving patient prognosis.

**Trial registration:**

The protocol of this study was approved by the Committee of Ethics from West China Hospital of Sichuan University (2019–473) with a waiver of informed consent and registered at www.chictr.org.cn (ChiCTR1900025160, 15/08/2019).

**Supplementary Information:**

The online version contains supplementary material available at 10.1186/s12877-024-05148-1.

## Background

Each year, 313 million surgeries are performed worldwide [1], and approximately one-third of elective surgeries are performed on patients over 65 years of age [2]. Surgery in geriatric patients often poses a risk of major postoperative complications because of age-related degenerative physiological characteristics [3].

Postoperative acute kidney injury (AKI) is a common major postoperative complication and is associated with both short-term and long-term adverse events, such as prolonged hospitalization, increased postoperative mortality, and further development of chronic kidney disease [4, 5]. Even mild kidney injury is related to increased morbidity and mortality [6]. The incidence of postoperative AKI varies between 2 and 30% depending on the study population and definition of AKI [7]. The in-hospital mortality rate for patients with postoperative AKI is 13.3%, compared with 0.9% for those without postoperative AKI [8]. Patients may not recover their initial state of renal function after the onset of kidney injury [9]. Therefore, it is critical to prevent the occurrence of postoperative AKI [10]. Early identification of patients at high risk of AKI could facilitate preventive measures and support perioperative management.

Existing risk assessment tools are mainly aimed at predicting postoperative AKI after cardiac surgery [11–13]. The prediction of AKI among noncardiac surgeries has been studied less extensively. Thorough evaluation is usually performed in patients with cardiac surgery because of the innate high postoperative AKI risk [14]. Patients with noncardiac surgery often have insufficient evaluation, and the probability of overlooking postoperative AKI is higher in noncardiac surgery than in cardiac surgery [15]. In fact, postoperative AKI results in an eightfold increased postoperative 30-day mortality for patients with noncardiac surgery [16]. It is necessary to identify risk factors and develop a model for predicting AKI following noncardiac surgery [7].

Several risk assessment tools have been developed for predicting AKI following noncardiac surgery. Due to several limitations, these tools have not been widely used in clinical settings. First, most prediction models were established by logistic regression [14]. Constraints on the logistic regression analysis method led the models to select risk factors among a small group of variables with presumed linear relationships, which may have contributed to the loss of potential predictors and reduction of predictive accuracy [17]. Second, although recent studies have demonstrated the potential of machine learning methods in predicting postoperative AKI [18, 19], the widespread application of machine learning models has been limited because they contain a large number of variables [20]. Third, existing models for predicting AKI following noncardiac surgery have often been restricted to specific surgery types [21, 22], so they lack generalizability to other surgical processes. In addition, no existing models have been developed for the specific assessment of geriatric patients. Compared with young patients, geriatric patients are more vulnerable to postoperative acute kidney injury (AKI) [3]. Geriatric patients constitute a specific population in medical research because of age-related degenerative physiological characteristics, and ignoring age categories can cause inaccurate parameter estimation. Previous studies have indicated that risk factors associated with postoperative AKI differ between younger and older populations [23]. Prediction models developed for general patient populations may not provide sufficient accuracy in geriatric patients [24].

In this study, we collected data prospectively and aimed to develop a simple machine learning model for predicting AKI following noncardiac surgery in geriatric patients, thus facilitating the clinical applicability of the machine learning model.

## Methods

### Data source

This study has been reported in line with the Strengthening The Reporting Of Cohort Studies in Surgery (STROCSS) criteria [25] and the Transparent Reporting of a Multivariable Prediction Model for Individual Prognosis or Diagnosis (TRIPOD) guidelines [26]. The data for the present study were obtained from a prospective cohort of geriatric patients built in a tertiary academic hospital in China from 2019. Patients aged ≥ 65 years who underwent noncardiac surgery between June 2019 and December 2021 were enrolled. Patients were excluded if they (1) had chronic kidney disease, defined as a preoperative estimated glomerular filtration rate lower than 60 ml × min^−1^ × 1.73 m^−2^ (ml·min^−1^·1.73 m^−2^) or a requirement for dialysis; (2) underwent urologic procedures; or (3) were lost to follow-up. If patients underwent multiple surgeries during the study period, only the first surgery was included in the analysis. Related patient data were collected by trained residents on the day before surgery. The attending physician and the resident rechecked the collected information before surgery. If any errors or omissions existed, the clinician would make corrections or supplement the information. Preoperative laboratory tests were automatically retrieved from the laboratory information system. All laboratory tests were performed within 7 days before surgery. If a patient had more than one result for the same test, the most recent preoperative result was used in the analysis. Preoperative clinical data included demographic characteristics, preoperative vital signs, laboratory tests and comorbidities.

To ascertain the presence of postoperative AKI, research personnel performed follow-up at 24 h postoperatively, 48 h postoperatively, before hospital discharge, and 7 days postoperatively. Patients who developed postoperative AKI were frequently contacted until recovery or death. Throughout each patient’s hospital stay, research personnel performed bedside follow-up visits; after hospital discharge, patients were contacted via phone.

### Outcome definition

The study outcome was the onset of postoperative AKI, defined using the diagnostic criteria in the Kidney Disease: Improving Global Outcomes study [27]. Specifically, postoperative AKI was defined by the presence of one of the following: creatinine elevation ≥ 26.5 μmol/L within 48 h postoperatively, compared with preoperative creatinine level; creatinine elevation ≥ 1.5-fold greater than baseline creatinine level within 7 days postoperatively; urine output < 0.5 ml/kg/h during the first 6h postoperatively. The preoperative serum creatinine value measured soon before surgery was regarded as the baseline creatinine level.

### Data preprocessing and model development

All variables are presented as continuous variables or categorical variables. Missing values were imputed by 0 s, with indicators representing missingness, which regarded missing values as a separate group. Data from June 2019 to March 2021 were used as training data, and data from April 2021 to December 2021 were used for internal validation. Training data were randomly split 80% for model training and 20% for model testing.

Univariate analysis was used to identify potential predictors of postoperative AKI in the training set. Every single factor at the *P* < 0.05 level was deemed statistically significant. The weight of evidence (WOE) [28] approach was used to discretize potential predictors, and weights were set for each category of each predictor. Then, we applied two algorithms for further feature selection, including the least absolute shrinkage and selection operator (LASSO) regularization algorithm and the random forest recursive feature elimination (RF-RFE) algorithm. In LASSO regression, features were selected according to the binomial deviance within one standard error of its minimum value. The RF-RFE method selected risk factors based on the area under the receiver operating characteristic curve (AUROC). Tenfold cross-validation was performed on the training set for parameter tuning.

Three classification methods were used to develop prediction models based on features selected by LASSO and RF-RFE, including LASSO, random forest (RF) and extreme gradient boosting (XGBoost). Parameter tuning was performed via grid search and tenfold cross-validation on the training set to construct prediction models. In LASSO regression, the classifier was trained with the L1 penalty, and the hyperparameter “max_iter” was used to constrain the model to avoid overfitting. In the RF and XGBoost models, we controlled the number of estimators and tree depth to avoid overfitting. The RF classifier was trained with 80 estimators, and the maximum tree depth was constrained to 4. The XGBoost classifier was trained by 60 estimators with a maximum tree depth of 3, and the learning rate was set at 0.3. The number of patients without postoperative AKI considerably outweighed the number of patients with postoperative AKI, which led to extreme class imbalance. To address this issue, the hyperparameter “class_weight” was set to “balanced” to automatically increase the weight of the positive sample in RF and LASSO, and “scale_pos_weight” was set to 1 in XGBoost. These hyperparameters were used for oversampling in the related model. Furthermore, we added clinically relevant predictors to the final model.

### Model evaluation

To evaluate the discrimination ability of the model, we calculated the sensitivity (recall), precision, F1 score, specificity, accuracy, area under the precision-recall curve (AUPRC), and AUROC. Among these performance metrics, precision and sensitivity can provide more direct insight into predictive performance when the class distribution is imbalanced [29]. The F1 score is the harmonic mean of precision and sensitivity. Compared with AUROC, AUPRC gives no credit for truly predicting negatives. For a model developed on an imbalanced dataset, AUPRC can give a more accurate interpretation of the model’s performance [29]. In this study, we chose the F1 score and AUPRC as the main evaluation metrics for model comparison. The Brier score was used to evaluate the calibration ability of the model. A lower Brier score value indicates better model performance (closer to 0 is ideal, and values > 0.3 indicate poor calibration) [30]. All model parameters were fixed after training the model on the training set. The best-performing model was further evaluated using the internal validation set. Sensitivity analysis was performed for the operation site.

### Model explanation

We used the SHAP algorithm [31] to elucidate the contribution of each predictor to the outcome predicted by the best-performing model and explain individual prediction. Shapley values were computed for all patients in the training and internal validation sets to measure overall variable importance and illustrated using a beeswarm plot.

The SHAP value of each feature can be calculated by a partial dependence plot (PDP or PD Plot) calculator, which can display the marginal effect of a single feature on the outcome predicted by the model [32]. A PDP can demonstrate whether a feature's relationship with an outcome is linear, monotonic, or more complex.

### Statistical analysis

Differences in variable distribution between training and internal validation sets were assessed for significance using Student's t test or Wilcoxon rank-sum test for numerical variables and chi-squared test or Fisher exact test for categorical variables. A 2-sided *P* value of < 0.05 was considered statistically significant. Bootstrapping was used on the test set and internal validation set to calculate the 95% confidence intervals. All statistical analyses were conducted via Python 3.7.6. Machine learning models were developed using the scikit-learn library.

## Results

### Patient characteristics

**Fig. 1:**
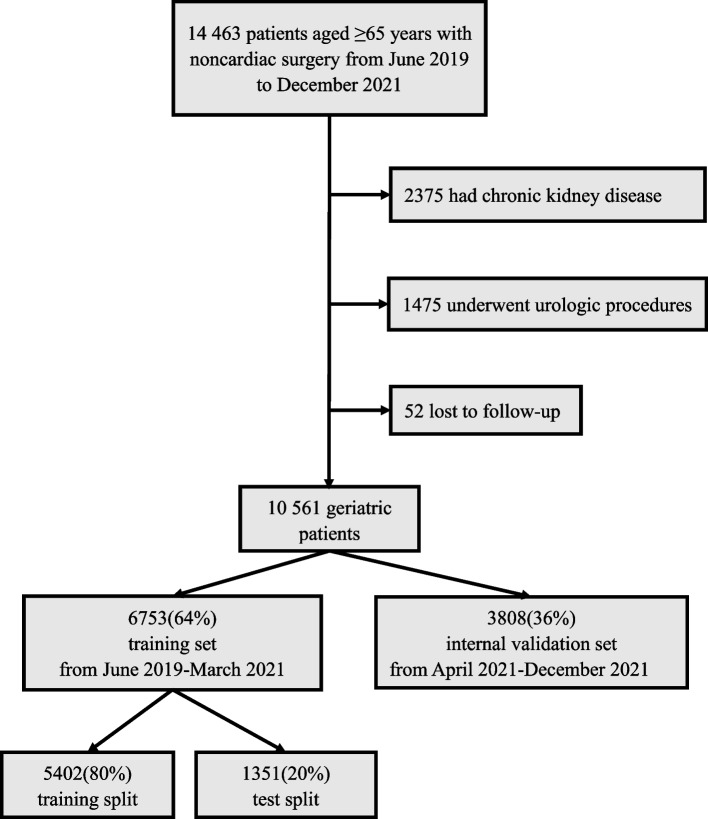
Flowchart for developing the training set and internal validation set

Of 14 463 geriatric patients with noncardiac surgery, a total of 3902 patients were excluded. The final dataset enrolled 10 561 geriatric patients, including 6753 patients in the training set (from June 2019 to March 2021) and 3808 patients in the internal validation set (from April 2021 to December 2021) (Fig. 1). The data distribution between these two sets was similar, despite statistical significance owing to the large sample size (Supplementary table S1). In the training dataset, 250 (3.70%) patients developed postoperative AKI. A smaller proportion of patients in the internal validation set suffered AKI (2.52%, P = 0.001 vs the training set).

### Feature selection and model comparison

Univariate analysis was used to identify potential predictors from 126 variables. The results showed that 69 variables were significantly associated with postoperative AKI (*P* < 0.05) (Supplementary table S2). In further feature selection, LASSO identified the eleven most influential predictors for discriminating postoperative AKI as λ increased to 0.006 (one standard error of the minimum λ) (Supplementary figure S1). The RF-RFE method achieved the highest AUROC when it included nine predictors (Supplementary figure S2).

LASSO, RF and XGBoost algorithms were used to develop prediction models based on features selected by LASSO and RF-RFE, respectively. The XGBoost model with RF-RFE selected features exhibited the highest AUPRC of 0.505 (95% confidence interval [CI]: 0.369–0.626) and the highest F1 score of 0.527 (95% CI: 0.385–0.659). For calibration, the Brier score of the XGBoost model was the lowest among all these models (0.025 [95%CI: 0.018–0.033]) (Table 1).

**Table 1:** Performance metrics of candidate models

Model (Feature selection method)	Precision (95%CI)	Sensitivity (95%CI)	F1 score (95%CI)	AUPRC (95%CI)	Specificity (95%CI)	AUROC (95%CI)	Brier score (95%CI)
Random forest (LASSO)	0.221 (0.148–0.290)	0.539 (0.404–0.667)	0.312 (0.222–0.391)	0.482 (0.352–0.607)	0.921 (0.906–0.935)	0.817 (0.752–0.877)	0.090 (0.083–0.096)
XGBoost (LASSO)	0.780 (0.600–0.923)	0.392 (0.265–0.531)	0.519 (0.379–0.652)	0.491 (0.362–0.616)	0.995 (0.991–0.998)	0.810 (0.741–0.873)	0.027 (0.020–0.035)
LASSO (LASSO)	0.105 (0.076–0.134)	0.779 (0.673–0.885)	0.185 (0.138–0.230)	0.452 (0.307–0.581)	0.722 (0.697–0.748)	0.823 (0.755–0.882)	0.168 (0.159–0.177)
Random forest (RF-RFE)	0.516 (0.365–0.667)	0.412 (0.280–0.550)	0.456 (0.325–0.581)	0.476 (0.340–0.598)	0.984 (0.976–0.991)	0.806 (0.736–0.869)	0.042 (0.036–0.049)
XGBoost (RF-RFE)	0.909 (0.769–1.000)	0.374 (0.246–0.509)	0.527 (0.385–0.659)	0.505 (0.369–0.626)	0.998 (0.996–1.000)	0.806 (0.733–0.875)	0.025 (0.018–0.033)
LASSO (RF-RFE)	0.103 (0.075–0.131)	0.779 (0.673–0.885)	0.182 (0.135–0.226)	0.414 (0.272–0.553)	0.716 (0.691–0.74)	0.812 (0.743–0.874)	0.171 (0.161–0.180)

*Abbreviations*: *CI* confidence interval, *AUPRC* area under the precision-recall curve, *AUROC* area under the receiver operating characteristic curve, *LASSO* least absolute shrinkage and selection operator regression, *XGBoost* extreme gradient boosting, *RF-RFE* random forest recursive feature elimination algorithm

**Fig. 2:**
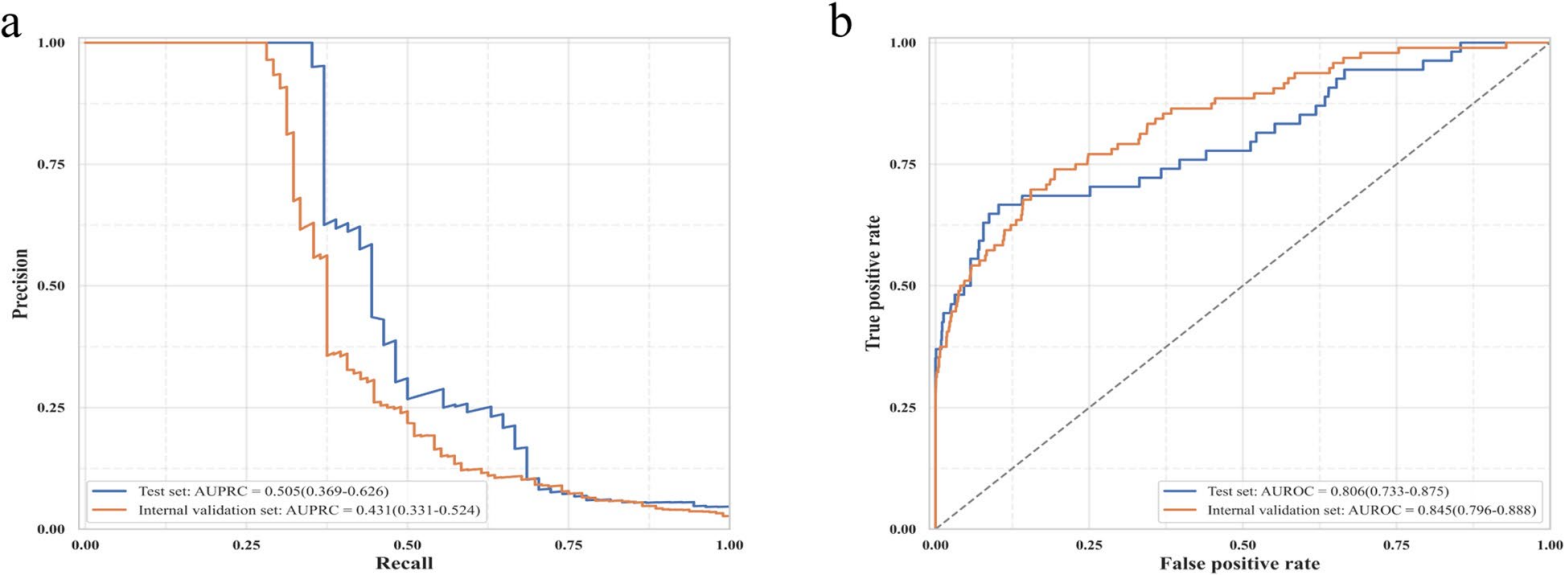
Performance characteristic curves of the final extreme gradient boosting model. **a** Precision-recall curves of the final extreme gradient boosting model based on the test set and internal validation set. **b** Receiver operating characteristic curves of the final extreme gradient boosting model based on the test set and internal validation set. Abbreviations: *AUPRC* area under the precision-recall curve, *AUROC* area under the receiver operating characteristic curve

Emergency surgery is widely considered to be associated with postoperative AKI [7, 33], so it was added to the final model. Predictors in the final model included hypertension, urine protein, diabetes mellitus, operation site, American Society of Anesthesiologists (ASA) classification, operation time, serum cystatin C level, coefficient of variation of red blood cell distribution width (RDW-CV), international normalized ratio (INR), and emergency surgery. In the internal validation, the final XGBoost model maintained good predictive performance, with an AUPRC of 0.431 (95%CI: 0.331–0.524) and an AUROC of 0.845 (95%CI: 0.796–0.888) (Fig. 2). Sensitivity analyses were performed on upper abdomen surgery, lower abdomen surgery and thoracic surgery. In the internal validation set, 852 patients underwent upper abdomen surgery, and 40 (4.69%) patients developed postoperative AKI. The final XGBoost model achieved an AUROC of 0.850 (95% CI: 0.777–0.912) and an AUPRC of 0.574 (95% CI: 0.419–0.714) (Supplementary figure S3). For lower abdomen surgery, 686 patients were enrolled, and 31 (4.52%) patients developed postoperative AKI. The final XGBoost model achieved an AUROC of 0.812 (95% CI: 0.717–0.896) and an AUPRC of 0.448 (95% CI: 0.276–0.619) (Supplementary figure S4). A total of 536 patients who underwent thoracic surgery were included, and 12 (2.24%) patients developed postoperative AKI. The final XGBoost model achieved an AUROC of 0.693 (95% CI: 0.506–0.866) and an AUPRC of 0.210 (95% CI: 0.026–0.470) (Supplementary figure S5).

### Model explanation

**Fig. 3:**
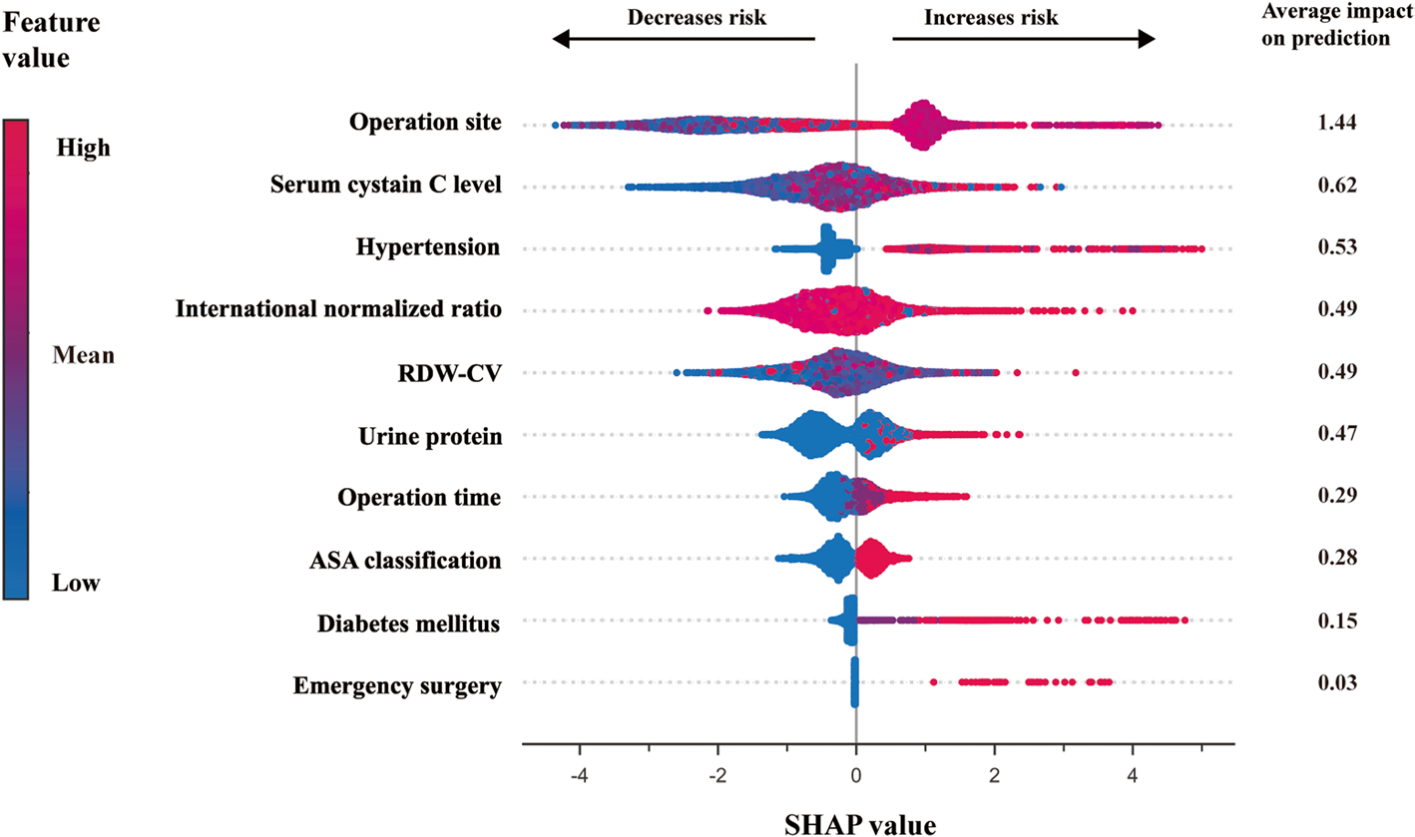
SHAP values of ten predictors incorporated in the final extreme gradient boosting model. Abbreviations: *RDW-CV* coefficient of variation of red blood cell distribution width, *ASA* American Society of Anesthesiologists, *SHAP* SHapley Additive exPlanations, *XGBoost* extreme gradient boosting

The ten predictors were subjected to the SHAP evaluator to acquire the contribution of each predictor to the prediction of the XGBoost model. Features with positive or negative Shapley values are correlated with higher or lower predicted risk for postoperative AKI, respectively. Blue indicates a decrease, and red indicates an increase in the indicated parameter. As presented in Fig. 3, all predictors had positive correlations with AKI.

**Fig. 4:**
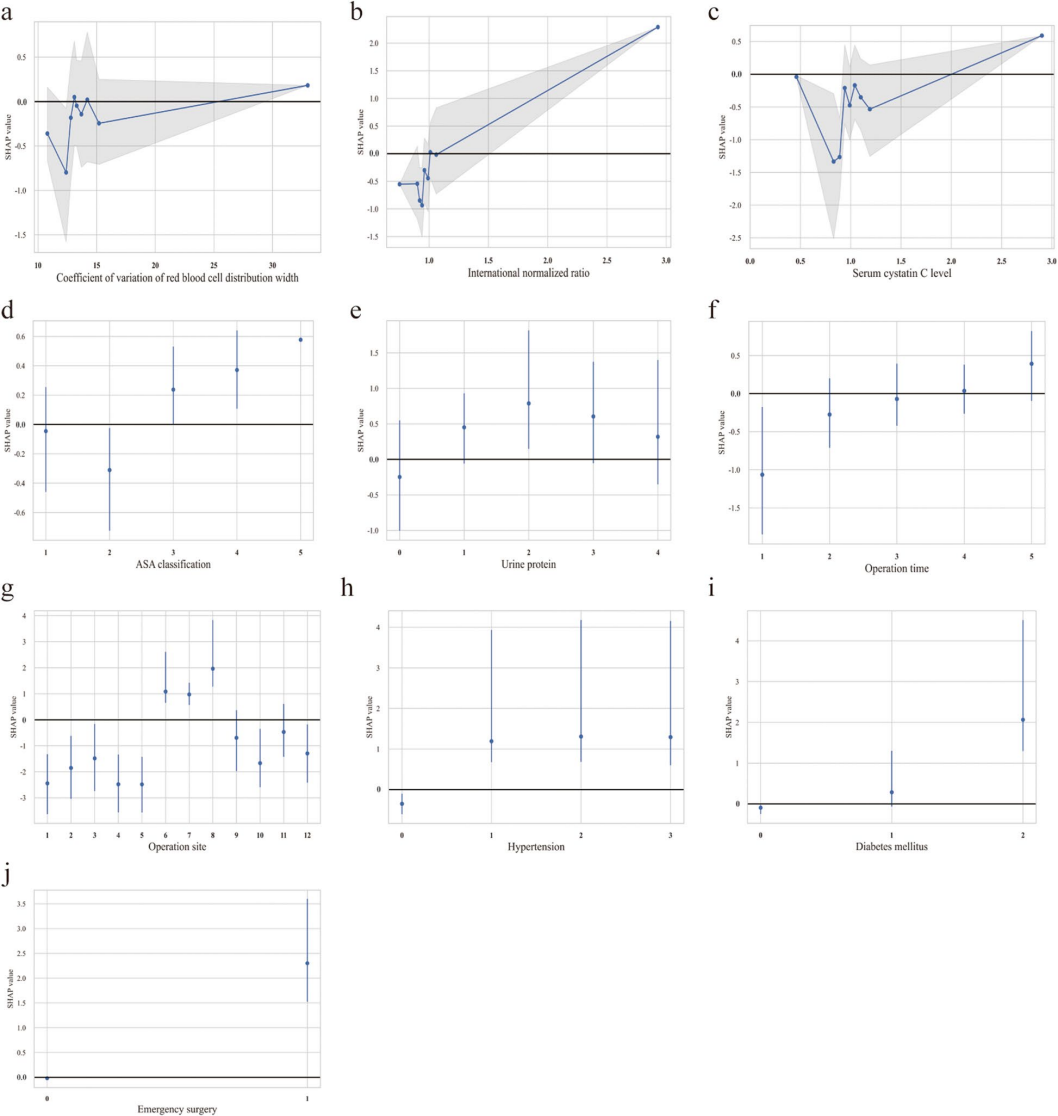
Partial dependence plots of predictors in the final prediction model. The actual value for each predictor is shown on the x-axis, and the SHAP value corresponding to the abscissa value is shown on the y-axis. Each point represents a patient sample in the database. Positive or negative SHAP values indicate which feature contributes to acute kidney injury (positive) or no acute kidney injury (negative). Abbreviations: *SHAP* SHapley Additive exPlanations, *ASA* American Society of Anesthesiologists

Numerical clinical parameters can change continuously, but the related risk may not increase or decrease linearly [32]. It is important to identify the threshold where the risk of predicted outcome abruptly changes. We used Shapley values to investigate how these specific features affected the predicted risk as the value was altered (Fig. 4). We found that the predicted risk of postoperative AKI increased at the following thresholds: RDW-CV > 25.5% (Fig. 4a), INR > 1.1 (Fig. 4b), and serum cystatin C level > 2 mg/L (Fig. 4c). For categorical parameters, predictors associated with increased risk of postoperative AKI included ASA classification higher than III (Fig. 4d), operation time ≥ 2 h (Fig. 4e), elevated urine protein (Fig. 4f), abdominal surgery (Fig. 4g), hypertension (Fig. 4h), diabetes mellitus (especially insulin-dependent diabetes mellitus) (Fig. 4i), and emergency surgery (Fig. 4j).

**Fig. 5:**
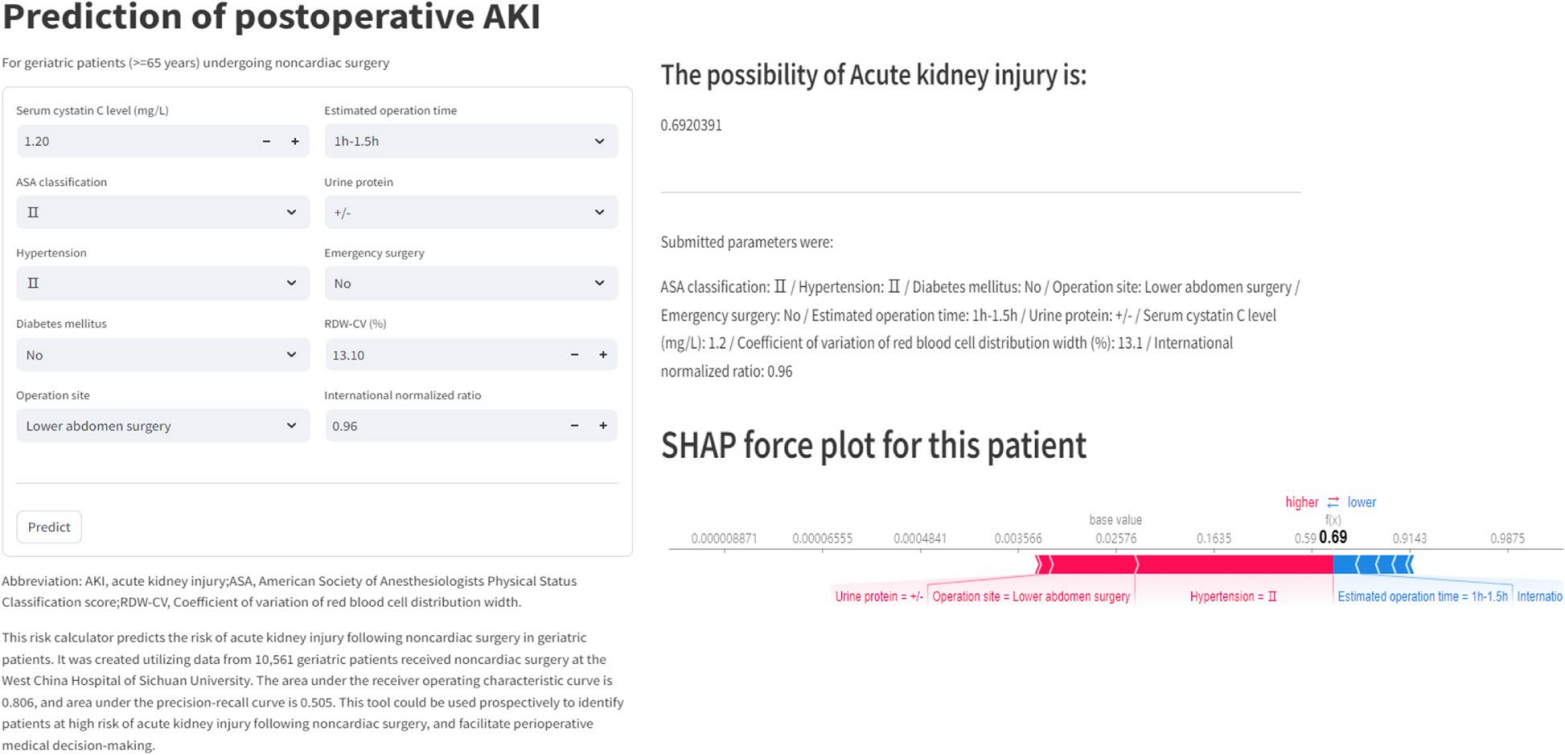
The postoperative acute kidney injury risk prediction for an example patient by the web calculator. The predicted probability of acute kidney injury was 0.69 for this patient, and related risk factors included grade II hypertension, lower abdomen surgery, and elevated urine protein

A web calculator was established for clinicians to use the model (available at https://huggingface.co/spaces/Yijie7/AKI_Prediction), and the interface of the calculator was shown in Fig. 5. The prediction result can be obtained after inputting the value of corresponding variable for the patient. For this patient, the predicted probability of AKI was 0.69, indicating that the patient was at high risk of AKI. Related risk factors included grade II hypertension, lower abdomen surgery, and elevated urine protein.

## Discussion

Postoperative AKI is associated with both short-term and long-term adverse events [4]. Accurate prediction of postoperative AKI risk can facilitate preoperative informed consent, perioperative medical decision-making, and resource utilization, thus improving patient prognosis. In this study, we collected data prospectively and developed a simple machine learning model for the preoperative prediction of AKI following noncardiac surgery in geriatric patients.

The transfer of complex machine learning models with numerous variables from research to real-world application poses additional challenges because of technical barriers, data security, and business considerations [20]. Considering the balance between predictive accuracy and ease of clinical application, we developed a simple XGBoost model using predictors selected by RF-RFE and made it available as a web calculator to facilitate greater application among clinicians. Victor J. Lei and colleagues developed machine learning models for predicting AKI following noncardiac surgery, and their model based on preoperative clinical data achieved an AUROC of 0.804 [19]. Our model achieved similar prediction performance with fewer predictors. Previous prediction models created by logistic regression analysis for predicting AKI following noncardiac surgery achieved AUROCs of 0.74 to 0.80 in the development set and 0.70 to 0.72 in the validation set [10, 14]. Temporal validation was conducted in our study, which simulates the practical application of the prediction model. Our model achieved a greater AUROC in the development set and maintained good predictive performance in the validation set. The results indicated that the machine learning model may be more robust than the model developed by logistic regression analysis.

A total of nine predictors were selected by the RF-RFE method and included in our final XGBoost model. For continuous variables, serum cystatin C, INR and RDW-CV were found to be strongly associated with postoperative AKI in our study. Serum cystatin C is eliminated solely via glomerular filtration and can be easily measured [34]. Compared with urea and creatinine, the serum cystatin C concentration increases earlier when the kidney is injured [35], and it is a useful predictor of short-term mortality and AKI in acute aortic dissection patients [36]. INR is a standardized measure of the extrinsic coagulation pathway. Elevated INR has been reported to be related to increased infection, bleeding, and mortality rates after total knee arthroplasty [37]. For patients undergoing liver transplantation, INR is found to be associated with postoperative AKI [38]. Red blood cell distribution width (RDW) reflects the variability in red blood cell size. Elevated RDW can be caused by erythrocyte production dysfunction or increased erythrocyte destruction [39]. Previous studies have demonstrated the value of RDW for predicting postoperative mortality and AKI [39, 40]. In our study, RDW-CV > 25.5%, INR > 1.1, or serum cystatin C level > 2 mg/L were found to be significantly associated with an increased risk of AKI following noncardiac surgery in geriatric patients.

Among categorical variables, we found that abdominal surgery, hypertension, elevated urine protein, operation time over 2 h, ASA classification higher than III, and diabetes mellitus (especially insulin-dependent diabetes mellitus) may lead to an increased risk of postoperative AKI in geriatric patients following noncardiac surgery. Abdominal surgery can cause elevated intra-abdominal pressure, which leads to mechanical compression of renal veins and constriction of renal arteries [41]. Patients who undergo abdominal surgery have a higher risk of renal hypoperfusion and subsequent onset of AKI [42]. Hypertension and diabetes mellitus have been broadly reported to be powerful predictors of postoperative AKI [15, 43]. The presence of urine protein can be an indicator of unrecognized glomerulonephritis, and preoperative urine protein has been reported to be independently associated with AKI following noncardiac surgery [44]. The ASA score evaluates patients’ physiological conditions based on the amount and severity of comorbidities, and it has been found to be closely related to postoperative AKI [22, 45]. In our prediction model, operation time and emergency surgery partially represent the clinical severity of the patient and surgery. Serum cystatin C and urine protein are closely related to underlying kidney function. The model incorporates the representative findings of patient comorbidities, clinical severity, baseline kidney function, and surgical difficulty in the preoperative period. We used actual operation time to identify the true relationship between operation time and postoperative AKI, and WOE was used to discretize continuous actual operation time into different categories. In the preoperative assessment, the operation time could be substituted by the estimated operation time.

As predictors incorporated in this model could be easily obtained during preoperative evaluation, the model could be used to identify high-risk geriatric patients before noncardiac surgery. Accurate preoperative prediction could facilitate the implementation of prophylactic interventions and the optimization of clinical resources. For example, timely fluid therapy and avoidance of nephrotoxic agents could be used to protect kidney function in high-risk patients during the perioperative period. In addition, our model may provide medical staff with "early warnings" through the analysis of important predictors. For geriatric patients with poorly controlled diabetes mellitus or hypertension, improving their preoperative health status may decrease the risk of AKI. Correcting the preoperative clotting status based on the INR and shortening the overall procedure may also be beneficial for high-risk patients.

Most prediction models were developed on combined data from geriatric patients and younger patients [10, 14]. However, geriatric patients constitute a specific population in medical research because of age-related changes in physiological characteristics, and risk factors associated with postoperative AKI differ between younger and older populations [23]. Ignoring age categories can cause inaccurate parameter estimation, so prediction models developed on general patient populations may be unsuitable for geriatric patients [24]. This study developed a prediction model based on data from geriatric patients to improve the prediction in geriatric patients.

This study has several limitations. First, we used data from a single institution to develop and internally validate our prediction model. Future studies are needed to verify the generalizability of our model to new datasets from other institutions. Second, unlike other measured actual values, the validity of estimated operation time is susceptible to subjective bias. Incorrect estimation may decrease the accuracy of the prediction result. Centers with limited availability of estimated operation time may find it difficult to use the risk prediction calculator. Third, limited by the small number of patients in each group (divided by operation site), sensitivity analyses were only performed on upper abdomen surgery, lower abdomen surgery and thoracic surgery. Our prediction model maintained good predictive ability in the sensitivity analyses for upper abdomen surgery and lower abdomen surgery and showed relatively poor performance for thoracic surgery. In the thoracic surgery subgroup, only twelve patients developed postoperative AKI. This result may not be accurate because of the small number of patients in this group. Future studies are needed to verify the predictive ability of our model for several subspecialties.

## Conclusions

In this study, we established a simple machine learning model based on easily available preoperative information for predicting AKI following noncardiac surgery in geriatric patients and made it available by developing a Web-based calculator. The accurate identification of patients with high postoperative AKI risk could facilitate preoperative informed consent, optimize perioperative decision-making, and aid in the allocation of medical resources.

### Supplementary Information

Supplementary Material 1.

Supplementary Material 2.

Supplementary Material 3.

Supplementary Material 4.

Supplementary Material 5.

Supplementary Material 6.

## Data Availability

The data that support the findings of this study are available on reasonable request from the corresponding author. The data are not publicly available due to privacy and ethical restrictions.

## Abbreviations

AKI: Acute kidney injury
STROCSS: Strengthening The Reporting Of Cohort Studies in Surgery
TRIPOD: Transparent Reporting of a Multivariable Prediction Model for Individual Prognosis or Diagnosis
WOE: Weight of evidence
LASSO: The least absolute shrinkage and selection operator
RF-RFE: Random forest recursive feature elimination
AUROC: Area under the receiver operating characteristic curve
RF: Random forest
XGBoost: Extreme gradient boosting
AUPRC: Area under the precision-recall curve
PDP or PD Plot: Partial dependence plot
ASA: American Society of Anesthesiologists
RDW-CV: Coefficient of variation of red blood cell distribution width
INR: International normalized ratio
RDW: Red blood cell distribution width

## References

[1] Nepogodiev D, Martin J, Biccard B, Makupe A, Bhangu A, Nepogodiev D (2019). Global burden of postoperative death. The Lancet.

[2] Kahli Z, Shelley RM, Richard S, Jeffrey B, Sandhya LD, Mitchell TH (2018). Preoperative Cognitive Impairment As a Predictor of Postoperative Outcomes in a Collaborative Care Model. JAGS.

[3] Oresanya LB, Lyons WL, Finlayson E (2014). Preoperative assessment of the older patient: a narrative review. JAMA.

[4] Shigehiko U, John AK, Rinaldo B, Gordon SD, Hiroshi M, Stanislao M (2005). Acute Renal Failure in Critically ill Patients. JAMA.

[5] Coca SG, Singanamala S, Parikh CR (2012). Chronic kidney disease after acute kidney injury: a systematic review and meta-analysis. Kidney Int.

[6] Bihorac A, Yavas S, Subbiah S, Hobson CE, Schold JD, Gabrielli A (2009). Long-term risk of mortality and acute kidney injury during hospitalization after major surgery. Ann Surg.

[7] Gumbert SD, Kork F, Jackson ML, Vanga N, Ghebremichael SJ, Wang CY (2020). Perioperative Acute Kidney Injury. Anesthesiology.

[8] O'Connor ME, Hewson RW, Kirwan CJ, Ackland GL, Pearse RM, Prowle JR (2017). Acute kidney injury and mortality 1 year after major non-cardiac surgery. Br J Surg.

[9] Steven CY, Lee HT (2012). Acute Kidney Injury and Extrarenal Organ Dysfunction. Anesthesiology.

[10] Bell S, Dekker FW, Vadiveloo T, Marwick C, Deshmukh H, Donnan PT (2015). Risk of postoperative acute kidney injury in patients undergoing orthopaedic surgery–development and validation of a risk score and effect of acute kidney injury on survival: observational cohort study. BMJ (Clinical research ed).

[11] Duminda NW, Keyvan K, Jean YD, Vivek R, Christopher TC, John TG (2007). Derivation and Validation of a Simplified Predictive Index for Renal Replacement Therapy After Cardiac Surgery. JAMA.

[12] Brown JR, Cochran RP, Leavitt BJ, Dacey LJ, Ross CS, MacKenzie TA (2007). Multivariable prediction of renal insufficiency developing after cardiac surgery. Circulation.

[13] Kate B, Veerle V, Domenico P, Moninder B, Kate T, Jonathan AS (2014). Predictive models for kidney disease: improving global outcomes (KDIGO) defined acute kidney injury in UK cardiac surgery. Crit Care.

[14] Park S, Cho H, Park S, Lee S, Kim K, Yoon HJ (2019). Simple Postoperative AKI Risk (SPARK) Classification before Noncardiac Surgery: A Prediction Index Development Study with External Validation. J Am Soc Nephrol.

[15] Grams ME, Sang Y, Coresh J, Ballew S, Matsushita K, Molnar MZ (2016). Acute Kidney Injury After Major Surgery: A Retrospective Analysis of Veterans Health Administration Data. Am J Kidney Dis.

[16] Kheterpal S, Tremper KK, Heung M, Rosenberg AL, Englesbe M, Shanks AM (2009). Development and validation of an acute kidney injury risk index for patients undergoing general surgery: Results from a national data set. Anesthesiology.

[17] Pirracchio R, Petersen ML, Carone M, Rigon MR, Chevret S, van der Laan MJ (2015). Mortality prediction in intensive care units with the Super ICU Learner Algorithm (SICULA): a population-based study. Lancet Respir Med.

[18] Rashidi P, Bihorac A (2020). Artificial intelligence approaches to improve kidney care. Nat Rev Nephrol.

[19] Lei VJ, Luong T, Shan E, Chen X, Neuman MD, Eneanya ND (2019). Risk Stratification for Postoperative Acute Kidney Injury in Major Noncardiac Surgery Using Preoperative and Intraoperative Data. JAMA Netw Open.

[20] Rank N, Pfahringer B, Kempfert J, Stamm C, Kuhne T, Schoenrath F (2020). Deep-learning-based real-time prediction of acute kidney injury outperforms human predictive performance. NPJ Digit Med.

[21] Ko S, Jo C, Chang CB, Lee YS, Moon YW, Youm JW (2022). A web-based machine-learning algorithm predicting postoperative acute kidney injury after total knee arthroplasty. Knee Surg Sports Traumatol Arthrosc.

[22] Chen Q, Zhang Y, Zhang M, Li Z, Liu J (2022). Application of Machine Learning Algorithms to Predict Acute Kidney Injury in Elderly Orthopedic Postoperative Patients. Clin Interv Aging.

[23] Saydy N, Mazine A, Stevens LM, Jeamart H, Demers P, Page P (2018). Differences and similarities in risk factors for postoperative acute kidney injury between younger and older adults undergoing cardiac surgery. J Thorac Cardiovasc Surg.

[24] Alrezk R, Jackson N, Al Rezk M, Elashoff R, Weintraub N, Elashoff D (2017). Derivation and Validation of a Geriatric-Sensitive Perioperative Cardiac Risk Index. J Am Heart Assoc.

[25] Mathew G and Agha R for the STROCSS Group. STROCSS (2021). Strengthening the Reporting of cohort, cross-sectional and case-control studies in Surgery. Int J Surg.

[26] Collins GS, Moons KGM, Dhiman P, Riley RD, Beam AL, Van Calster B (2024). TRIPOD+AI statement: updated guidance for reporting clinical prediction models that use regression or machine learning methods. BMJ (Clinical research ed).

[27] Khwaja A (2012). KDIGO clinical practice guidelines for acute kidney injury. Nephron Clin Pract.

[28] Mironchyk P, Tchistiakov V. Monotone optimal binning algorithm for credit risk modeling. Financ Econ. 2017:1–15. 10.13140/RG.2.2.31885.44003.

[29] Saito T, Rehmsmeier M (2015). The precision-recall plot is more informative than the ROC plot when evaluating binary classifiers on imbalanced datasets. PLoS ONE.

[30] Staffa SJ, Zurakowski D (2021). Statistical Development and Validation of Clinical Prediction Models. Anesthesiology.

[31] Lundberg SM, Erion G, Chen H, DeGrave A, Prutkin JM, Nair B (2020). From Local Explanations to Global Understanding with Explainable AI for Trees. Nat Mach Intell.

[32] Jiang Z, Bo L, Xu Z, Song Y, Wang J, Wen P (2021). An explainable machine learning algorithm for risk factor analysis of in-hospital mortality in sepsis survivors with ICU readmission. Comput Methods Programs Biomed.

[33] Goren O, Matot I (2015). Perioperative acute kidney injury. Br J Anaesth..

[34] Spahillari A, Parikh CR, Sint K, Koyner JL, Patel UD, Edelstein CL (2012). Serum Cystatin C– Versus Creatinine-Based Definitions of Acute Kidney Injury Following Cardiac Surgery: A Prospective Cohort Study. Am J Kidney Dis.

[35] Shlipak MG, Matsushita K, Ärnlöv J, Inker LA, Katz R, Polkinghorne KR (2013). Cystatin C versus creatinine in determining risk based on kidney function. N Engl J Med.

[36] Wang J, Yang B, Liu M, You T, Shen H, Chen Y (2022). Serum cystatin C is a potential predictor of short-term mortality and acute kidney injury in acute aortic dissection patients: a retrospective cohort study. J Thorac Dis.

[37] Rudasill SE, Liu J, Kamath AF (2019). Revisiting the International Normalized Ratio (INR) Threshold for Complications in Primary Total Knee Arthroplasty: An Analysis of 21,239 Cases. J Bone Joint Surg Am.

[38] Guo M, Gao Y, Wang L, Zhang H, Liu X, Zhang H (2020). Early Acute Kidney Injury Associated with Liver Transplantation: A Retrospective Case-Control Study. Med Sci Monit..

[39] Duchnowski P, Hryniewiecki T, Kuśmierczyk M, Szymański P (2020). Anisocytosis predicts postoperative renal replacement therapy in patients undergoing heart valve surgery. Cardiol J.

[40] Chan DXH, Sim YE, Chan YH, Poopalalingam R, Abdullah HR (2018). Development of the Combined Assessment of Risk Encountered in Surgery (CARES) surgical risk calculator for prediction of postsurgical mortality and need for intensive care unit admission risk: a single-center retrospective study. BMJ Open.

[41] Dalfino L, Tullo L, Donadio I, Malcangi V, Brienza N (2008). Intra-abdominal hypertension and acute renal failure in critically ill patients. Intensive Care Med.

[42] Demarchi AC, de Almeida CT, Ponce D, e Castro MC, Danaga AR, Yamaguti FA (2014). Intra-abdominal pressure as a predictor of acute kidney injury in postoperative abdominal surgery. Ren Fail..

[43] Wang Y, Bellomo R (2017). Cardiac surgery-associated acute kidney injury: risk factors, pathophysiology and treatment. Nat Rev Nephrol.

[44] Nishimoto M, Murashima M, Kokubu M, Matsui M, Eriguchi M, Samejima K-I (2020). Pre-operative proteinuria and post-operative acute kidney injury in noncardiac surgery: the NARA-Acute Kidney Injury cohort study. Nephrol Dial Transplant.

[45] Hawkins J, Mpody C, Corridore M, Cambier G, Tobias JD, Nafiu OO (2022). Risk Factors and Consequences of Acute Kidney Injury After Noncardiac Surgery in Children. Anesth Analg.

