# Supplementary figures and images for: A simple machine learning model for the prediction of acute kidney injury following noncardiac surgery in geriatric patients: a prospective cohort study
Source: BMC Geriatr. 2024 Jun 25;24:549. doi: 10.1186/s12877-024-05148-1 (PMC11197315; doi:10.1186/s12877-024-05148-1)

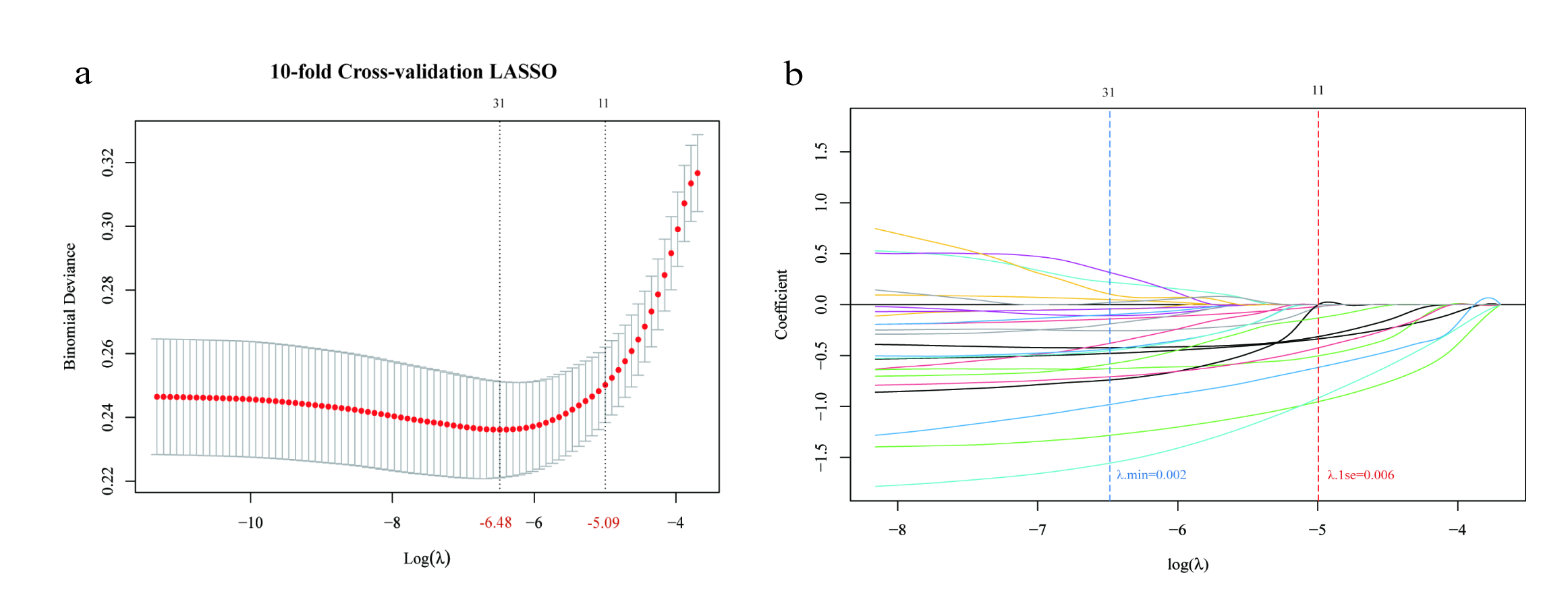

Supplement: Supplementary file 1 — Supplementary Material 1. [file 12877_2024_5148_MOESM1_ESM.tif]

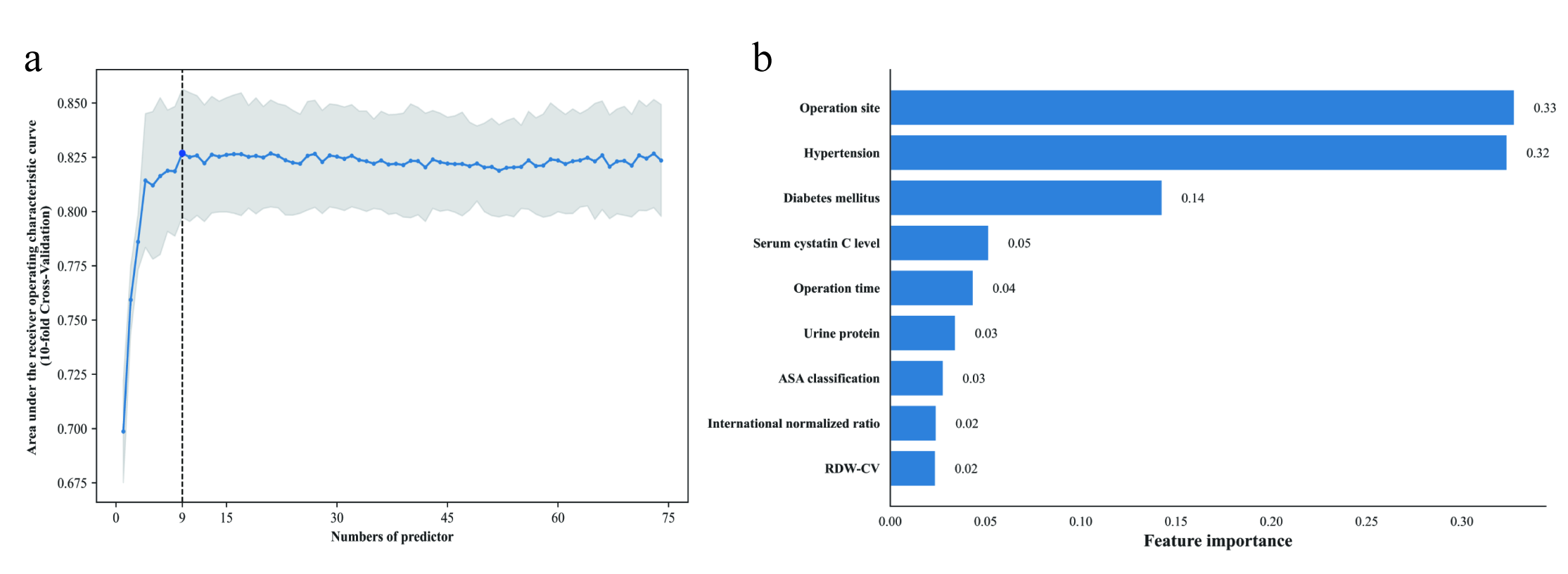

Supplement: Supplementary file 2 — Supplementary Material 2. [file 12877_2024_5148_MOESM2_ESM.tif]

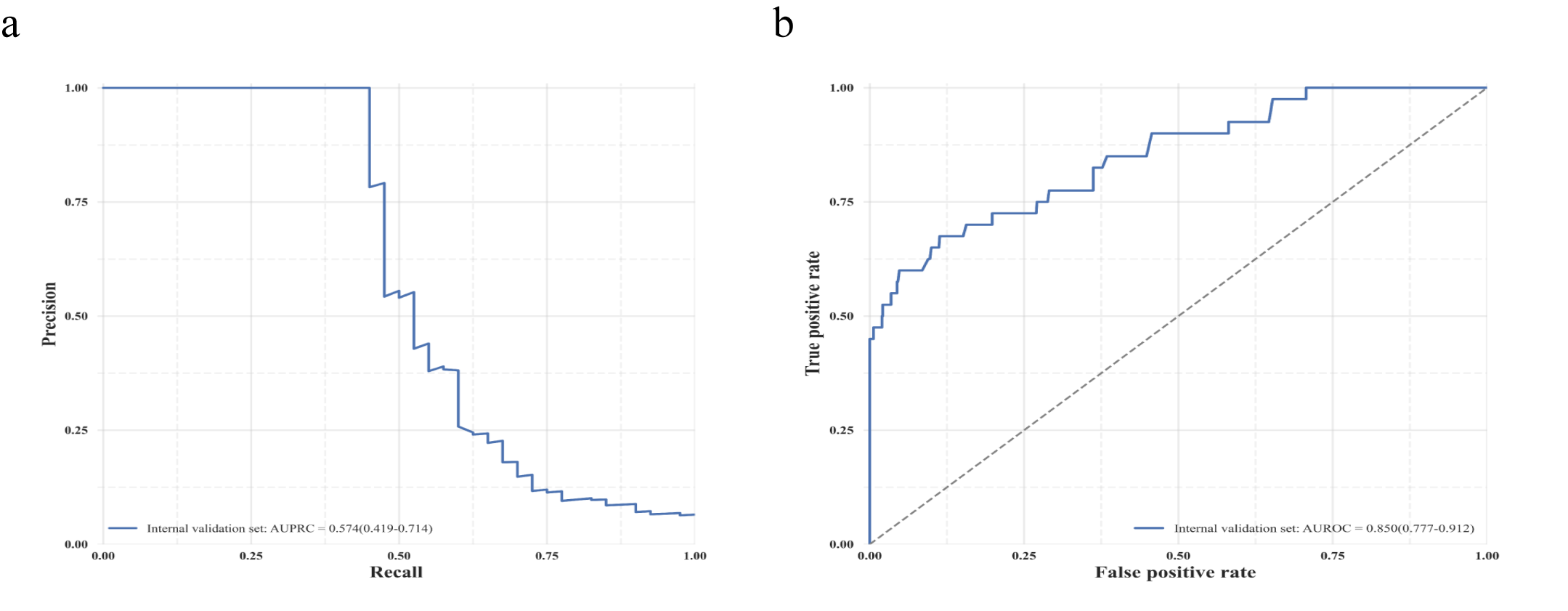

Supplement: Supplementary file 3 — Supplementary Material 3. [file 12877_2024_5148_MOESM3_ESM.tif]

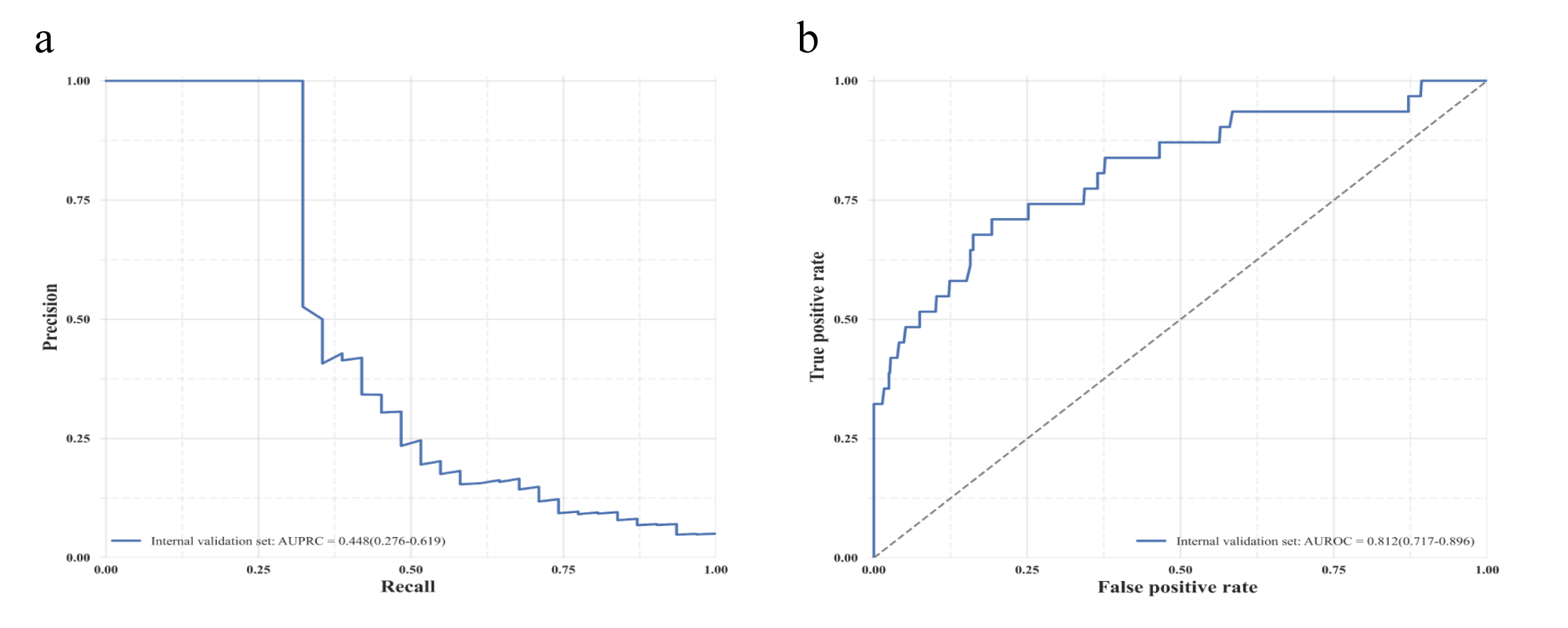

Supplement: Supplementary file 4 — Supplementary Material 4. [file 12877_2024_5148_MOESM4_ESM.tif]

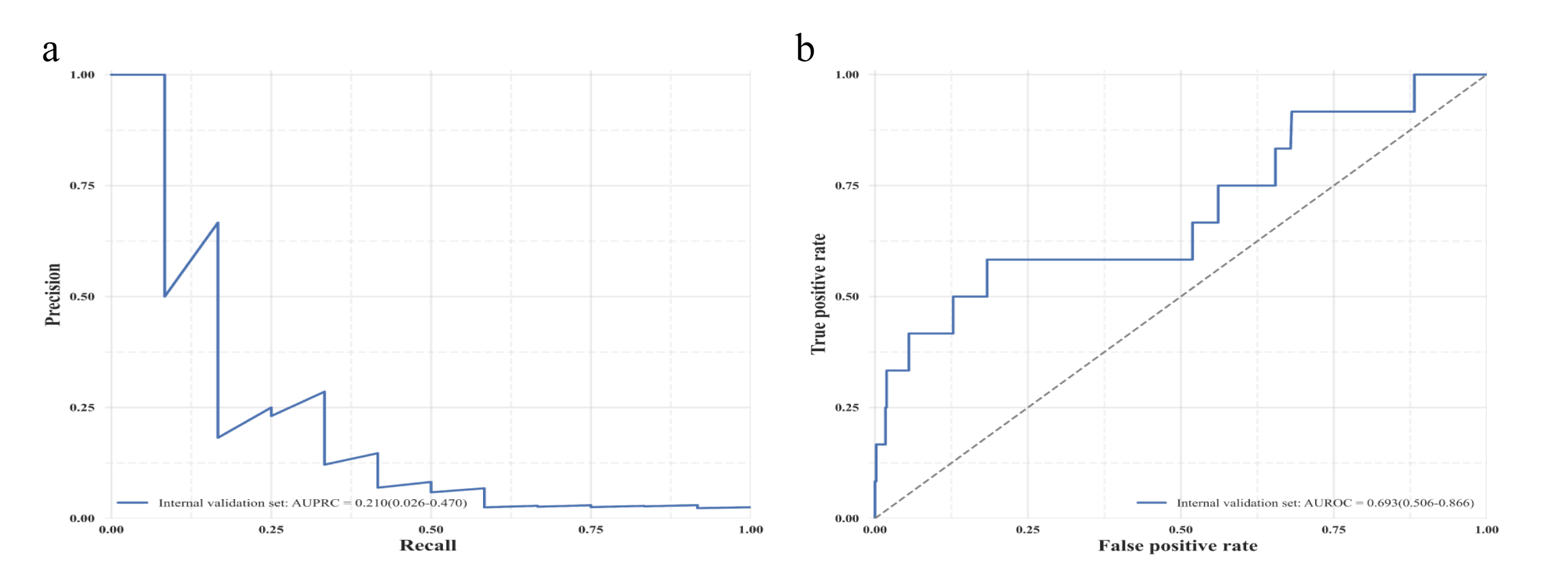

Supplement: Supplementary file 5 — Supplementary Material 5. [file 12877_2024_5148_MOESM5_ESM.tif]
