# Supplementary material for: A simple machine learning model for the prediction of acute kidney injury following noncardiac surgery in geriatric patients: a prospective cohort study
Source: BMC Geriatr. 2024 Jun 25;24:549. doi: 10.1186/s12877-024-05148-1 (PMC11197315; doi:10.1186/s12877-024-05148-1)
Supplement: Supplementary file 6 — Supplementary Material 6. [file 12877_2024_5148_MOESM6_ESM.docx]

**A simple machine learning model for the prediction of acute kidney injury following noncardiac surgery in geriatric patients: A prospective cohort study-Supplementary file**

Supplementary Table S1. Patients’ characteristics and incidences of postoperative acute kidney injury in the training and internal validation sets.

| Variable | Training set  (n=6753) | Internal validation  set (n=3808) | *P* value |
| --- | --- | --- | --- |
| Male | 3563(52.8) | 1981(52.0) | 0.465 |
| Age (years) | 70(67-74) | 69(67-74) | 0.596 |
| BMI (kg/m^2^) | 23.2(21.1-25.4) | 23.2(21.0-25.4) | 0.794 |
| Smoking | 849(12.6) | 442(11.6) | 0.146 |
| Alcohol | 177(2.6) | 97(2.6) | 0.819 |
| Systolic blood pressure (mmHg) | 133(122-145) | 133(121-145) | 0.983 |
| Diastolic blood pressure (mmHg) | 80(73-87) | 80(74-88) | 0.051 |
| Respiratory rate (/min) | 20(19-20) | 20(19-20) | 0.001 |
| Heart rate (/min) | 79(71-87) | 79(71-86) | 0.831 |
| Body temperature (℃) | 36.5(36.2-36.5) | 36.4(36.3-36.6) | <0.001 |
| ASA classification |  |  | <0.001 |
| Ⅰ | 15(0.2) | 0(0) |  |
| Ⅱ | 3742(55.4) | 2277(59.8) |  |
| Ⅲ | 2975(44.1) | 1519(39.9) |  |
| IV | 20(0.3) | 12(0.3) |  |
| V | 1(0.01) | 0(0) |  |
| NYHA classification |  |  | <0.001 |
| Ⅰ | 3893(57.7) | 1896(49.8) |  |
| Ⅱ | 2583(38.3) | 1779(46.7) |  |
| Ⅲ | 163(2.4) | 99(2.6) |  |
| IV | 9(0.1) | 3(0.1) |  |
| Functional capacity |  |  | <0.001 |
| >6MET | 1040(15.4) | 372(9.8) |  |
| 3-6MET | 4462(66.1) | 2853(74.9) |  |
| <3MET | 1091(16.2) | 510(13.4) |  |
| the FRAIL Scale |  |  | 0.862 |
| fit | 6613(97.9) | 3725(97.8) |  |
| pre-frail | 103(1.5) | 59(1.6) |  |
| frail | 37(0.6) | 24(0.6) |  |
| General condition |  |  | 0.005 |
| partial dependence | 1974(29.2) | 1008(26.5) |  |
| dependence | 82(1.2) | 33(0.9) |  |
| Conscious state |  |  | 0.781 |
| sober | 6741(99.8) | 3799(99.7) |  |
| somnolence | 4(0.06) | 2(0.05) |  |
| light coma | 1(0.01) | 1(0.03) |  |
| deep coma | 1(0.01) | 0(0) |  |
| Operation site |  |  | <0.001 |
| dermatological surgery | 486(7.2) | 257(6.8) |  |
| limb surgery | 398(5.9) | 208(5.5) |  |
| arthroplasty | 912(13.5) | 516(13.6) |  |
| spinal surgery | 396(5.9) | 279(7.3) |  |
| head and neck surgery | 653(9.7) | 365(9.6) |  |
| upper abdomen surgery | 1389(20.6) | 852(22.4) |  |
| lower abdomen surgery | 1498(22.2) | 686(18.0) |  |
| abdomen surgery | 31(0.5) | 18(0.5) |  |
| thoracic surgery | 852(12.6) | 536(14.1) |  |
| cranial surgery | 9(0.1) | 12(0.3) |  |
| thoracoabdominal surgery | 50(0.7) | 30(0.8) |  |
| vascular surgery | 71(1.1) | 41(1.1) |  |
| Operation time (min) | 100(65-170) | 102(65-170) | 0.216 |
| Emergency surgery | 23(0.3) | 18(0.5) | 0.295 |
| Open surgery | 4885(72.3) | 3283(86.2) | <0.001 |
| Endoscopic surgery | 311(4.6) | 208(5.5) | 0.050 |
| Mallampati airway classification |  |  | <0.001 |
| I | 1251(17.8) | 413(10.5) |  |
| Ⅱ | 4940(73.2) | 3159(83.0) |  |
| Ⅲ | 527(7.8) | 228(6.0) |  |
| IV | 35(0.5) | 8(0.2) |  |
| COPD |  |  | <0.001 |
| stable | 267(3.9) | 89(2.3) |  |
| exacerbations | 6(0.1) | 3(0.1) |  |
| repeated exacerbations within 1 year≥3 | 4(0.1) | 1(0.03) |  |
| Respiratory infection within last 1 month | 91(1.4) | 28(0.7) | 0.004 |
| Asthma | 59(0.9) | 38(1.0) | 0.521 |
| Obstructive sleep apnea | 1381(20.5) | 646(17.0) | <0.001 |
| Pneumothorax | 2(0.03) | 0(0) | 0.539 |
| Pleural effusion | 17(0.3) | 21(0.6) | 0.014 |
| Hypertension |  |  | 0.191 |
| Ⅰ | 421(6.2) | 236(6.2) |  |
| Ⅱ | 550(8.1) | 357(9.4) |  |
| Ⅲ | 323(4.8) | 176(4.6) |  |
| Ischemic heart disease |  |  | 0.490 |
| no chest pain within 3 months | 140(2.1) | 89(2.3) |  |
| chest pain within 3 months | 26(0.4) | 21(0.6) |  |
| acute myocardial infarction >3 months | 10(0.2) | 3(0.1) |  |
| acute myocardial infarction <3 months | 2(0.03) | 1(0.03) |  |
| Valvular heart disease | 14(0.2) | 0(0) | 0.005 |
| Dilated cardiomyopathy | 10(0.2) | 0(0) | 0.017 |
| Hypertrophic cardiomyopathy |  |  | 0.525 |
| non-obstractive | 2(0.03) | 2(0.05) |  |
| obstractive | 2(0.03) | 0(0) |  |
| Congestive heart failure | 2(0.03) | 2(0.05) | 0.623 |
| Arrhythmia | 348(5.2) | 130(3.4) | <0.001 |
| Artery disease | 27(0.4) | 13(0.3) | 0.639 |
| Venous disease | 19(0.3) | 10(0.3) | 0.860 |
| Stroke | 100(1.5) | 54(1.4) | 0.796 |
| Transient ischemic attack | 51(0.8) | 23(0.6) | 0.371 |
| Intracranial hypertension | 1(0.01) | 0(0) | 1.000 |
| Cerebral hernia | 2(0.03) | 0(0) | 0.539 |
| Neuromuscular disease | 28(0.4) | 11(0.3) | 0.306 |
| Psychiatric disorder | 39(0.6) | 24(0.6) | 0.735 |
| Diabetes mellitus |  |  | 0.752 |
| non-insulin dependent | 119(1.8) | 71(1.9) |  |
| insulin dependent | 167(2.5) | 102(2.7) |  |
| Thyroid dysfunction |  |  | 0.573 |
| hyperthyroidism | 23(0.3) | 9(0.2) |  |
| hypothyroidism | 89(1.3) | 46(1.2) |  |
| Liver disease | 218(3.2) | 95(2.5) | 0.033 |
| Liver cirrhosis | 55(0.8) | 38(1.0) | 0.333 |
| Ascites | 1(0.01) | 0(0) | 1.0 |
| Lower digestive tract hemorrhage within last 1 week | 36(0.5) | 25(0.7) | 0.422 |
| Upper digestive tract hemorrhage within last 1 week | 9(0.1) | 8(0.2) | 0.344 |
| Gastroesophageal reflux | 77(1.1) | 42(1.1) | 0.862 |
| Peptic ulcer | 79(1.2) | 33(0.9) | 0.144 |
| Immunity dysfunction | 2(0.03) | 1(0.03) | 1.000 |
| Autoimmune disease | 21(0.3) | 19(0.5) | 0.131 |
| Vasoactive medications | 5(0.1) | 3(0.1) | 1.000 |
| Anti-heart failure drugs | 5(0.1) | 4(0.1) | 0.731 |
| Anticoagulants and antiplatelet agents | 145(2.2) | 108(2.8) | 0.026 |
| Glucocorticoid | 17(0.3) | 10(0.3) | 0.915 |
| Hemoglobin (g/L) | 131(120-141) | 132(121-142) | 0.281 |
| Hematocrit (L/L) | 0.40(0.37-0.43) | 0.40(0.37-0.43) | 0.830 |
| Red blood cell count (10^12^/L) | 4.29(3.93-4.64) | 4.32(3.97-4.67) | 0.004 |
| MCH (pg) | 30.8(29.6-31.9) | 30.7(29.5-31.8) | 0.009 |
| MCHC (g/L) | 327(320-333) | 328(322-335) | <0.001 |
| MCV (fL) | 93.9(90.8-97.0) | 93.2(90.3-96.1) | <0.001 |
| RDW-CV (%) | 13.3(12.8-14.2) | 13.3(12.8-14.2) | 0.093 |
| RDW-SD (fL) | 46.0(43.7-48.8) | 45.4(43.0-48.3) | <0.001 |
| Blood platelet count (10^9^/L) | 175(136-218) | 175(137-220) | 0.615 |
| White blood cell count (10^9^/L) | 5.62(4.67-6.76) | 5.59(4.60-6.73) | 0.120 |
| Neutrophil granulocyte percentage (%) | 60.9(54.2-67.8) | 60.7(54.2-67.6) | 0.333 |
| Neutrophil granulocyte count (10^9^/L) | 3.35(2.63-4.29) | 3.34(2.59-4.27) | 0.127 |
| Monocyte percentage (%) | 7.7(6.5-9.1) | 7.9(6.6-9.3) | <0.001 |
| Monocyte count (10^9^/L) | 0.43(0.34-0.54) | 0.44(0.35-0.55) | 0.058 |
| Basophil percentage (%) | 0.6(0.4-0.8) | 0.5(0.4-0.8) | 0.142 |
| Basophil count (10^9^/L) | 0.03(0.02-0.04) | 0.03(0.02-0.04) | 0.025 |
| Acidophil percentage (%) | 2.2(1.3-3.5) | 2.1(1.2-3.5) | 0.055 |
| Acidophil count (10^9^/L) | 0.12(0.07-0.20) | 0.12(0.07-0.19) | 0.009 |
| Lymphocyte percentage (%) | 27.6(21.0-33.8) | 27.6(21.5-33.7) | 0.498 |
| Lymphocyte count (10^9^/L) | 1.49(1.15-1.89) | 1.49(1.16-1.88) | 0.933 |
| APTT (s) | 27.2(25.8-28.9) | 26.5(25.2-27.9) | <0.001 |
| Prothrombin time (s) | 10.7(10.3-11.3) | 10.7(10.3-11.2) | 0.466 |
| Thrombin time (s) | 17.9(17.3-18.7) | 17.46(16.9-18.0) | <0.001 |
| INR | 0.96(0.92-1.01) | 0.97(0.93-1.02) | <0.001 |
| Fibrinogen (g/L) | 2.92(2.50-3.50) | 2.96(2.53-3.52) | 0.055 |
| Alanine aminotransferase (IU/L) | 16(12-24) | 17(12-26) | 0.196 |
| Aspartate aminotransferase (IU/L) | 20(17-26) | 20(17-26) | 0.560 |
| AST/ALT | 1.23(0.98-1.56) | 1.22(0.96-1.56) | 0.452 |
| Direct bilirubin (umol/L) | 3.1(2.3-4.2) | 3.2(2.5-4.3) | <0.001 |
| Indirect bilirubin (umol/L) | 7.9(6.1-10.5) | 8.2(6.3-10.7) | <0.001 |
| Total bilirubin (umol/L) | 11.2(8.7-14.7) | 11.5(9.1-15.1) | <0.001 |
| Total bile acid (umol/L) | 4.4(2.6-7.5) | 4.3(2.5-7.6) | 0.675 |
| Albumin (g/L) | 42.1(39.4-44.6) | 42.6(39.9-45.0) | <0.001 |
| Globulin (g/L) | 25.8(23.2-28.6) | 25.7(22.9-28.5) | 0.030 |
| Albumin-globulin ratio | 1.63(1.45-1.83) | 1.66(1.47-1.86) | <0.001 |
| Total protein (g/L) | 68.1(63.8-72.1) | 68.4(64.3-72.1) | 0.043 |
| Creatinine (umol/L) | 72(61-82) | 71(61-81) | 0.082 |
| e-GFR (ml/min/1.73m^2^) | 84.37(74.82-90.90) | 85.36(75.79-91.01) | 0.028 |
| Serum cystatin C level (mg/L) | 0.98(0.88-1.09) | 1.01(0.91-1.12) | <0.001 |
| Blood urea (mmol/L) | 5.3(4.4-6.3) | 5.3(4.5-6.3) | 0.071 |
| Uric acid (mmol/L) | 301(252-354) | 301(253-355) | 0.579 |
| Cholesterol (mmol/L) | 4.51(3.86-5.18) | 4.59(3.90-5.27) | 0.002 |
| Triglyceride (mmol/L) | 1.19(0.89-1.62) | 1.20(0.89-1.69) | 0.228 |
| Low density lipoprotein (mmol/L) | 2.67(2.12-3.23) | 2.74(2.16-3.32) | 0.001 |
| High density lipoprotein (mmol/L) | 1.24(1.03-1.49) | 1.29(1.07-1.55) | <0.001 |
| Glutamyl transpeptidase (IU/L) | 20(14-33) | 21(14-34) | 0.061 |
| Lactate dehydrogenase (IU/L) | 169(149-194) | 176(155-202) | <0.001 |
| Alkaline phosphatase (IU/L) | 79(66-97) | 81(67-100) | <0.001 |
| Hydroxybutyrate dehydrogenase (IU/L) | 131(115-150) | 131(115-151) | 0.522 |
| Blood β-hydroxybutyrate (mmol/L) | 0.09(0.07-0.14) | 0.10(0.07-0.15) | <0.001 |
| Creatine kinase (IU/L) | 75(53-103) | 78(57-108) | <0.001 |
| Blood glucose (mmol/L) | 5.17(4.76-5.80) | 5.19(4.78-5.86) | 0.080 |
| Serum sodium (mmol/L) | 141.4(139.9-142.7) | 141.1(139.6-142.4) | <0.001 |
| Serum chlorine (mmol/L) | 104.0(102.3-105.7) | 104.3(102.5-106.0) | <0.001 |
| Serum calcium (mmol/L) | 2.25(2.18-2.32) | 2.25(2.18-2.32) | 0.300 |
| Serum magnesium (mmol/L) | 0.89(0.85-0.94) | 0.90(0.85-0.94) | <0.001 |
| Serum potassium (mmol/L) | 4.00(3.78-4.23) | 4.00(3.78-4.21) | 0.543 |
| Serum phosphorus (mmol/L) | 1.07(0.95-1.19) | 1.08(0.96-1.20) | 0.003 |
| Anion gap (mmol/L) | 16.7(15.0-18.4) | 16.7(15.1-18.4) | 0.682 |
| Epithelium (/uL) | 3(1-6) | 2(1-6) | 0.003 |
| Urine specific gravity | 1.02(1.01-1.02) | 1.02(1.01-1.03) | <0.001 |
| Urobilinogen (umol/L) |  |  | 0.110 |
| 1+ | 123(1.8) | 75(2.0) |  |
| 2+ | 15(0.2) | 19(0.5) |  |
| 3+ | 3(0.04) | 1(0.03) |  |
| 4+ | 2(0.03) | 0(0) |  |
| Urine bilirubin (umol/L) |  |  | 0.286 |
| 1+ | 5(0.07) | 0(0) |  |
| 2+ | 3(0.04) | 0(0) |  |
| 3+ | 7(0.10) | 4(0.11) |  |
| Urine glucose (mmol/L) |  |  | 0.273 |
| +/- | 65(1.0) | 48(1.3) |  |
| 1+ | 37(0.6) | 31(0.8) |  |
| 2+ | 44(0.7) | 30(0.8) |  |
| 3+ | 40(0.6) | 18(0.5) |  |
| 4+ | 26(0.4) | 12(0.3) |  |
| Urine protein (mg/L) |  |  | 0.023 |
| +/- | 454(6.7) | 214(5.6) |  |
| 1+ | 184(2.7) | 77(2.0) |  |
| 2+ | 54(0.8) | 26(0.7) |  |
| 3+ | 7(0.1) | 3(0.1) |  |
| Urine ketone (mmol/L) |  |  | 0.160 |
| 1+ | 26(0.4) | 14(0.4) |  |
| 2+ | 12(0.2) | 1(0.03) |  |
| 3+ | 5(0.1) | 1(0.03) |  |
| 4+ | 1(0.01) | 0(0) |  |
| SpO_2_ (%) | 98(97-99) | 98(96-99) | <0.001 |
| CO_2_CP (mmol/L) | 24.5(22.8-26.2) | 23.96(22.4-25.6) | <0.001 |
| Postoperative acute kidney injury | 250(3.70) | 96(2.52) | 0.001 |

Footnote: Data are median (interquartile range) or n (%). Abbreviations: *BMI* body mass index, *ASA* American Society of Anesthesiologists, *NYHA* New York Heart Association, *MET* metabolic equivalent, *COPD* chronic obstructive pulmonary disease, *MCH* mean corpuscular hemoglobin, *MCHC* mean corpuscular hemoglobin concentration, *MCV* mean corpuscular volume, *RDW-CV* coefficient of variation of red blood cell distribution width, *RDW-SD* standard deviation of red blood cell distribution width, *APTT* activated partial thromboplastin time, *INR* international normalized ratio, *AST* activated partial thromboplastin time, *ALT* alanine aminotransferase, *e-GFR* estimated glomerular filtration rate, *SpO_2_* oxygen saturation, *CO_2_CP* Carbon dioxide combining power.

Supplementary Table S2. Potential predictors identified by univariate analysis.

| Variable | No acute kidney injury (n=6503) | Acute kidney injury (n=250) | *P* value |
| --- | --- | --- | --- |
| Male | 3399(52.3) | 164(65.6) | <0.001 |
| Age (years) | 69(67-74) | 71(67-76) | 0.001 |
| Alcohol | 165(2.5) | 12(4.8) | 0.028 |
| Heart rate (/min) | 78(71-87) | 80(73-89) | 0.008 |
| ASA classification |  |  | <0.001 |
| Ⅰ | 13(0.2) | 2(0.8) |  |
| Ⅱ | 3658(56.3) | 84(33.6) |  |
| Ⅲ | 2814(43.3) | 161(64.4) |  |
| IV | 18(0.3) | 2(0.8) |  |
| V | 0(0) | 1(0.4) |  |
| NYHA classification |  |  | <0.001 |
| Ⅰ | 3785(58.2) | 108(43.2) |  |
| Ⅱ | 2466(37.9) | 117(46.8) |  |
| Ⅲ | 145(2.2) | 18(7.2) |  |
| IV | 8(0.1) | 1(0.4) |  |
| Functional capacity |  |  | 0.001 |
| >6MET | 1015(15.6) | 25(10.0) |  |
| 3-6MET | 4305(66.2) | 157(62.8) |  |
| <3MET | 1032(15.9) | 59(23.6) |  |
| the FRAIL Scale |  |  | <0.001 |
| fit | 6377(98.1) | 236(94.4) |  |
| pre-frail | 95(1.5) | 8(3.2) |  |
| frail | 31(0.5) | 6(2.4) |  |
| General condition |  |  | <0.001 |
| partial dependence | 1882(28.9) | 92(36.8) |  |
| dependence | 72(1.1) | 10(4.0) |  |
| Operation site |  |  | <0.001 |
| dermatological surgery | 484(7.4) | 2(0.8) |  |
| limb surgery | 391(6.0) | 7(2.8) |  |
| arthroplasty | 892(13.7) | 20(8.0) |  |
| spinal surgery | 393(6.0) | 3(1.2) |  |
| head and neck surgery | 651(10.0) | 2(0.8) |  |
| upper abdomen surgery | 1294(19.9) | 95(38.0) |  |
| lower abdomen surgery | 1411(21.7) | 87(34.8) |  |
| abdomen surgery | 23(0.4) | 8(3.2) |  |
| thoracic surgery | 831(12.8) | 21(8.4) |  |
| cranial surgery | 9(0.1) | 0(0) |  |
| thoracoabdominal surgery | 48(0.7) | 2(0.8) |  |
| vascular surgery | 69(1.1) | 2(0.8) |  |
| Operation time (min) | 100(65-169) | 151(95-233) | <0.001 |
| Emergency surgery | 18(0.3) | 5(2.0) | 0.001 |
| Endoscopic surgery | 293(4.5) | 18(7.2) | 0.046 |
| COPD |  |  | 0.001 |
| stable | 251(3.9) | 16(6.4) |  |
| exacerbations | 5(0.1) | 1(0.4) |  |
| repeated exacerbations within 1 year≥3 | 2(0.03) | 2(0.8) |  |
| Pleural effusion | 11(0.2) | 6(2.4) | <0.001 |
| Hypertension |  |  | <0.001 |
| Ⅰ | 394(6.1) | 27(10.8) |  |
| Ⅱ | 505(7.8) | 45(18.0) |  |
| Ⅲ | 294(4.5) | 29(11.6) |  |
| Ischemic heart disease |  |  | 0.003 |
| no chest pain within 3 months | 127(2.0) | 13(5.2) |  |
| chest pain within 3 months | 25(0.4) | 1(0.4) |  |
| acute myocardial infarction >3 months | 8(0.1) | 2(0.8) |  |
| acute myocardial infarction <3 months | 2(0.03) | 0(0) |  |
| Dilated cardiomyopathy | 8(0.1) | 2(0.8) | 0.050 |
| Arrhythmia | 324(5.0) | 24(9.6) | 0.001 |
| Artery disease | 23(0.4) | 4(1.6) | 0.016 |
| Stroke | 92(1.4) | 8(3.2) | 0.031 |
| Diabetes mellitus |  |  | <0.001 |
| non-insulin dependent | 111(1.7) | 8(3.2) |  |
| insulin dependent | 137(2.1) | 30(12.0) |  |
| Liver cirrhosis | 48(0.7) | 7(2.8) | 0.004 |
| Ascites | 0(0) | 1(0.4) | 0.037 |
| Vasoactive medications | 1(0.02) | 4(1.6) | <0.001 |
| Anticoagulants and antiplatelet agents | 130(2.0) | 15(6.0) | <0.001 |
| Hemoglobin (g/L) | 132(120-142) | 126(110-139) | <0.001 |
| Hematocrit (L/L) | 0.40(0.37-0.43) | 0.39(0.34-0.43) | <0.001 |
| Red blood cell count (10^12^/L) | 4.29(3.93-4.64) | 4.18(3.70-4.64) | 0.015 |
| MCH (pg) | 30.8(29.6-31.9) | 30.4(28.9-31.7) | 0.002 |
| MCHC (g/L) | 327(320-333) | 326(318-332) | 0.020 |
| MCV (fL) | 93.9(90.8-97.0) | 92.9(89.6-96.4) | 0.011 |
| RDW-CV (%) | 13.3(12.8-14.2) | 13.7(13.1-15.1) | <0.001 |
| RDW-SD (fL) | 46.0(43.7-48.7) | 47.0(44.1-50.4) | <0.001 |
| White blood cell count (10^9^/L) | 5.61(4.66-6.74) | 5.86(4.84-7.21) | 0.029 |
| Neutrophil granulocyte percentage (%) | 60.9(54.1-67.7) | 64.1(56.6-71.2) | <0.001 |
| Neutrophil granulocyte count (10^9^/L) | 3.34(2.63-4.27) | 3.67(2.82-4.79) | <0.001 |
| Monocyte percentage (%) | 7.7(6.5-9.1) | 8.0(6.8-9.7) | 0.005 |
| Monocyte count (10^9^/L) | 0.43(0.34-0.54) | 0.47(0.38-0.59) | <0.001 |
| Acidophil count (10^9^/L) | 0.12(0.07-0.20) | 0.14(0.08-0.21) | 0.021 |
| Lymphocyte percentage (%) | 27.7(21.1-34.0) | 24.5(17.9-30.6) | <0.001 |
| Lymphocyte count (10^9^/L) | 1.49(1.15-1.89) | 1.34(0.98-1.79) | 0.001 |
| APTT (s) | 27.2(25.8-28.8) | 27.6(26.0-29.6) | 0.012 |
| Prothrombin time (s) | 10.7(10.3-11.3) | 11.0(10.4-11.8) | <0.001 |
| Thrombin time (s) | 18.0(17.3-18.7) | 17.8(17.1-18.7) | 0.011 |
| INR | 0.96(0.91-1.01) | 0.99(0.93-1.05) | <0.001 |
| Fibrinogen (g/L) | 2.92(2.50-3.47) | 3.17(2.65-3.89) | <0.001 |
| Direct bilirubin (umol/L) | 3.1(2.3-4.2) | 3.5(2.3-5.3) | <0.001 |
| Total bile acid (umol/L) | 4.3(2.5-7.4) | 5.1(2.8-9.2) | 0.011 |
| Albumin (g/L) | 42.1(39.4-44.7) | 40.8(37.1-43.5) | <0.001 |
| Albumin-globulin ratio | 1.63(1.45-1.83) | 1.58(1.37-1.75) | <0.001 |
| Total protein (g/L) | 68.1(63.9-72.1) | 67.1(62.7-71.5) | 0.016 |
| Creatinine (umol/L) | 71(61-82) | 76(63-85) | 0.011 |
| Serum cystatin C level (mg/L) | 0.98(0.88-1.08) | 1.07(0.95-1.20) | <0.001 |
| Cholesterol (mmol/L) | 4.53(3.87-5.19) | 4.12(3.39-4.84) | <0.001 |
| Low density lipoprotein (mmol/L) | 2.68(2.14-3.24) | 2.38(1.72-2.98) | <0.001 |
| High density lipoprotein (mmol/L) | 1.24(1.03-1.49) | 1.09(0.86-1.34) | <0.001 |
| Alkaline phosphatase (IU/L) | 79(66-96) | 85(67-111) | 0.002 |
| Glutamyl transpeptidase (IU/L) | 20(14-33) | 24(16-50) | <0.001 |
| Hydroxybutyrate dehydrogenase (IU/L) | 131(115-150) | 127(111-149) | 0.049 |
| Creatine kinase (IU/L) | 75(54-103) | 63(45-91) | <0.001 |
| Blood glucose (mmol/L) | 5.17(4.76-5.79) | 5.31(4.81-6.33) | 0.004 |
| Serum sodium (mmol/L) | 141.4(139.9-142.7) | 141.1(138.9-142.6) | 0.042 |
| Serum calcium (mmol/L) | 2.25(2.18-2.32) | 2.24(2.16-2.31) | 0.007 |
| Serum phosphorus (mmol/L) | 1.07(0.95-1.19) | 1.04(0.93-1.15) | 0.009 |
| Serum magnesium (mmol/L) | 0.89(0.85-0.94) | 0.87(0.82-0.92) | <0.001 |
| Urine specific gravity | 1.02(1.01-1.02) | 1.02(1.01-1.03) | 0.027 |
| Urobilinogen (umol/L) |  |  | 0.004 |
| 1+ | 113(1.7) | 10(4.0) |  |
| 2+ | 13(0.2) | 2(0.8) |  |
| 3+ | 2(0.03) | 1(0.4) |  |
| 4+ | 2(0.03) | 0(0) |  |
| Urine glucose (mmol/L) |  |  | <0.001 |
| +/- | 60(0.9) | 5(2.0) |  |
| 1+ | 31(0.5) | 6(2.4) |  |
| 2+ | 42(0.7) | 2(0.8) |  |
| 3+ | 36(0.6) | 4(1.6) |  |
| 4+ | 23(0.4) | 3(1.2) |  |
| Urine protein (mg/L) |  |  | <0.001 |
| +/- | 428(6.6) | 26(10.4) |  |
| 1+ | 165(2.5) | 19(7.6) |  |
| 2+ | 47(0.7) | 7(2.8) |  |
| 3+ | 6(0.1) | 1(0.4) |  |

Footnote: Data are median (interquartile range) or n (%). Abbreviations: *ASA* American Society of Anesthesiologists, *NYHA* New York Heart Association, *MET* metabolic equivalent, *COPD* chronic obstructive pulmonary disease, *MCH* mean corpuscular hemoglobin, *MCHC* mean corpuscular hemoglobin concentration, *MCV* mean corpuscular volume, *RDW-CV* coefficient of variation of red blood cell distribution width, *RDW-SD* standard deviation of red blood cell distribution width, *APTT* activated partial thromboplastin time, *INR* international normalized ratio.

Figure legends

Supplementary Figure S1. Feature selection using LASSO in the training set. (a) LASSO-based logistic model with 10-fold cross-validation based on the minimal binomial deviance was preformed to select feature. The binomial deviance vs log (λ) is shown in the plot. Two dotted vertical lines show the optimal λ values based, one on the criterion of minimal binomial deviance (λmin), and the other on one standard error of the minimum (λ1se). The log (λ1se) of -5.09 and λ1se of 0.006 were considered optimal. (b) LASSO coefficient profile of all variables against the log (λ) sequence. Dotted vertical lines in blue and red correspond to log(λmin) and log(λ1se). Eleven predictors with non-zero coefficients were identified according to the log (λ1se) value. Abbreviations: *LASSO* least absolute shrinkage and selection operator regression.

Supplementary Figure S2. Feature selection based on the RF-RFE method with 10-fold cross-validation in the training set. (a) The RF-RFE model for feature selection had the highest area under the receiver operating characteristic curve when it included nine predictors. (b) Relative importance of nine confirmed predictors. Abbreviations: *ASA* American Society of Anesthesiologists, *RDW-CV* coefficient of variation of red blood cell distribution width, *RF-RFE* random forest recursive feature elimination algorithm.

Supplementary Figure S3. Performance characteristic curves of the final extreme gradient boosting model in patients with upper abdomen surgery. (a) Precision-recall curves of the final extreme gradient boosting model based on the internal validation set. (b) Receiver operating characteristic curves of the final extreme gradient boosting model based on the internal validation set. Abbreviations: *AUPRC* area under the precision-recall curve, *AUROC* area under the receiver operating characteristic curve.

Supplementary Figure S4. Performance characteristic curves of the final extreme gradient boosting model in patients with lower abdomen surgery. (a) Precision-recall curves of the final extreme gradient boosting model based on the internal validation set. (b) Receiver operating characteristic curves of the final extreme gradient boosting model based on the internal validation set. Abbreviations: *AUPRC* area under the precision-recall curve, *AUROC* area under the receiver operating characteristic curve.

Supplementary Figure S5. Performance characteristic curves of the final extreme gradient boosting model in patients with thoracic surgery. (a) Precision-recall curves of the final extreme gradient boosting model based on the internal validation set. (b) Receiver operating characteristic curves of the final extreme gradient boosting model based on the internal validation set. Abbreviations: *AUPRC* area under the precision-recall curve, *AUROC* area under the receiver operating characteristic curve.
